# From Talent Identification to *Novo Basquete Brasil* (NBB): Multifactorial Analysis of the Career Progression in Youth Brazilian Elite Basketball

**DOI:** 10.3389/fpsyg.2021.617563

**Published:** 2021-03-16

**Authors:** Dilson B. Ribeiro Junior, Francisco Z. Werneck, Hélder Z. Oliveira, Patrícia S. Panza, Sergio J. Ibáñez, Jeferson M. Vianna

**Affiliations:** ^1^Faculty of Physical Education and Sports, Federal University of Juiz de Fora, Juiz de Fora, Brazil; ^2^Sports School, Federal University of Ouro Preto, Ouro Preto, Brazil; ^3^Salgado de Oliveira University, Juiz de Fora, Brazil; ^4^Faculty of Sport Sciences, University of Extremadura, Cáceres, Spain

**Keywords:** relative age effect, career progression, sports talent, basketball, talent identification, talent development

## Abstract

This study examined individual, task, and environmental constraints that influence the career progression of youth Brazilian elite basketball players and the probability of reaching *Novo Basquete Brasil* (NBB) and to determine if the association of the relative age effect (RAE) is a key factor in the career progression. The sample consisted of 4,692 male players who were registered to participate in at least one U15, U17, or U22 youth Brazilian basketball championship between 2004 and 2018. Athletes who reached a high-performance level were coded like NBB players (9.6%). The birthdates, height, body mass, playing position, geographic region, club, competition category, and team performance were retrieved from the official data archive of the Brazilian Basketball Confederation and the National Basketball League. The maturity status was estimated using the predicted age at peak height velocity. A binary logistic regression examined the influence of each characteristic on the probability of a youth Brazilian basketball player to reach the NBB. The receiver operating characteristic (ROC) curves and the associated area under the curve (AUC) were used to assess the discriminant ability of the model. The taller and younger players not selected early into national teams, without specialization by playing position, who participated in U22 national championship, migrated to the southeast region, and remained in the formation process over time have a greater chance to reach the NBB. The ROC curve demonstrated an AUC of 93%. A combination of individual, task, and environmental characteristics influences the sport career of a young Brazilian basketball player in reaching the NBB. Further, early-maturing athletes have a greater chance to reach higher performances. RAE influences lower-level categories, but not a “NBB player's” career progression. The coaches, stakeholders, and practitioners should perform a holistic evaluation of sport talent in terms of a constraint-based theoretical model with the aim of avoiding bias produced by the maturational status and RAE in the youth Brazilian elite basketball.

## Introduction

The talent identification and development in sport is a dynamic process where individual, task, and environmental constraints interact in predicting long-term success (Phillips et al., 2010; Rees et al., 2016). Coaches and researchers are interested in identifying talented players (the most promising young athletes with the greatest sport potential) and how to optimize the long-term nurture in talent development programs in order for an athlete to become an expert—senior elite performance (Gulbin et al., 2013; Fransen and Gullich, 2019). The talent pathway is a key concern for sporting organizations and funding agencies due to considerable time and resource investments (Punkhurst and Collins, 2013). To date, there has been scientific evidence highlighting the necessity of longitudinal studies with multidisciplinary approaches for better understanding of how a career progression can be influenced from talent to expert, especially in team sports (Fransen and Gullich, 2019).

In basketball, performance and success are multifactorial; and many aspects, such as anthropometric, physiological, technical, tactical, psychological, and environmental, are required to become an elite athlete (Sáenz-López et al., 2005). In cross-sectional and short-term longitudinal studies, stature (Zarić et al., 2020), birthdate (Torres-Unda et al., 2013; Rubajczyk et al., 2017), maturation (Arede et al., 2019), physical fitness (Hoffman et al., 1996; Hoare, 2000), and skills (Arede et al., 2019) can be useful to predict individual performance and the selection of young basketball athletes. Thus, a more holistic and ecological analysis is considered an advance in the talent identification and development process to provide additional insight in the sport potential of the players (Moxley and Towne, 2015; Ribeiro Júnior et al., 2019). Hence, young athletes who present the right combination of characteristics required for good performance in basketball will probably have a greater chance of success. However, selecting athletes in the initial stage of talent development only from the physical performance perspective typically favors older members within a cohort, especially when in combination with early maturation and the influence of relative age effect (RAE) phenomenon (Pearson et al., 2006; Cobley et al., 2009; Rubajczyk et al., 2017).

The RAE is an immediate and long-term consequence of differences in chronological age between athletes who compete in the same age category. This phenomenon can be observed by an overrepresentation of players who are born closest to the cutoff date of the selection year (Cobley et al., 2009). The advantage in body size and physical performance of older and early-maturing players may confound the potential assessment of the player and could result in potential talent loss (Cripps et al., 2016). The coaches must to be aware that selection in the youth team sports tends to have a RAE impact (especially a maturational gradient), favoring those who have temporary advantages in relation to the others. In general, athletes who do not present outstanding performance in early phases of talent development are not recognized as a talent and do not receive the necessary support in order to develop their full potential (Votteler and Höner, 2014). However, maturation is not the only RAE explanation. The constraint-based theoretical model has been proposed to explain causes and consequences of the RAE considering the interaction between the aspects related to individual, task, and environment factors (Wattie et al., 2015).

Moreover, studies based on career progression have investigated if RAE is a factor that may influence the achievement of sport career success (de la Rubia et al., 2020). According to the highlighted results presented in de la Rubia et al. (2020) study, there is a RAE impact in short-term individual and team performances; however, in spite of RAE, the reverse was observed in the long-term competition performance. The RAE presence in basketball was confirmed in youth athletes (Torres-Unda et al., 2013, 2016; Arrieta et al., 2016; Rubajczyk et al., 2017) associated with performance (Ibáñez et al., 2018). In professional players, this phenomenon is less consistent (Werneck et al., 2016; Subijana and Lorenzo-Calvo, 2018; Lupo et al., 2019; Oliveira et al., 2019).

In Brazilian basketball, there is a player selection bias with respect to chronological age in the early stages. The RAE has been found from U12 to U22, in different geographic regions, playing position, and it is associated with team performance and stature (Oliveira et al., 2017; Ribeiro Júnior, 2020; Ribeiro Júnior et al., 2020). In addition, RAE is evident in the early senior career of players who reached the *Novo Basquete Brasil* (NBB); nevertheless, it disappears and even reverts (RAE reversal) in high performance (Oliveira et al., 2019), although there is no evidence of the RAE impact in the Brazilian basketball players related to the career progression (from talent, sport potential, to the expert, high level).

Furthermore, contextual factors such RAE (Cobley et al., 2009; de la Rubia et al., 2020), birthplace (Côté et al., 2006; Baker et al., 2009) and qualitative and quantitative practice (Ford et al., 2009; Moesch et al., 2011; Rees et al., 2016) must be considered in the expertise development. Some studies have demonstrated that birthplace is more important than birthdate effects on the achievement of sporting expertise (Côté et al., 2006; Baker et al., 2009). In basketball, high levels of proficiency and selection are correlated with an earlier start in sport and a later specialization (Leite and Sampaio, 2012; Arede et al., 2019). Retrospectively, research with the Chinese (Bonal et al., 2020) and the Brazilian elite basketball players (Cunha et al., 2017; Beneli, 2018) found relevant contextual factors to talent development pathway.

Recent research in basketball has attempted to track the development of young talented athletes into adulthood. Youth success, specialization, and birthdate, for example, do not appear to predict late success at the elite level (Barreiros et al., 2014; Güllich and Emrich, 2014). Professional players emerged from repeated procedures of selection and deselection (Güllich, 2014). However, a small percentage of athletes recognized as talented from the Spain national basketball youth teams reached senior performance (Sáenz-López et al., 2006; Ibáñez et al., 2010) improving from junior national teams to professional athletes (Sáenz-López et al., 2006; Subijana and Lorenzo-Calvo, 2018). In European youth national basketball teams, the re-selection process is influenced by the initial selection age, inverse RAE, and the country long-term performance (Kalén et al., 2020).

The basketball is a cultural and traditional sport in Brazil, which achieved significant international results in the 20th century (Beneli, 2018). Since the second decade of the 21st century, the Brazilian basketball has recovered its international representativeness. The following events were important for this recovery: (1) the exportation of a significant number of players to the major basketball leagues worldwide; (2) the establishment (in 2008) of the national basketball league [organized by clubs with the Brazilian Basketball Confederation (CBB) seal], which organizes the main professional adult Championship—NBB; and (3) the improvement of international results in the competitive scenario by the national team, Brazilian clubs, and youth national teams.

According to the CBB, Brazil has over 3 million basketball practitioners, over 1,000 teams across the country, 31 million fans, and 13 million super fans, which create the context of Brazilian basketball as an “open sea” for the player development opportunity. At the same time, there is little organization in regard to development and monitoring of the young basketball players in Brazil, from early development stages to achieve the high level of competition (Ribeiro Júnior, 2020). It is possible to highlight some efforts by the scientific community in the characterization of high-level senior players, as well as the training and development process of young Brazilian basketball players (Cunha et al., 2017; Beneli et al., 2020). Subsequently, it will be a long haul for the sports science community to better understand the path of the Brazilian basketball long-term development of youth basketball players in Brazil.

Despite previous studies, it is necessary to investigate factors that influence the development of the athlete from talent to expert level within a multifactorial, longitudinal, and constraint-based approach to better understand how the career progression in youth Brazilian basketball players occurs. According to our knowledge, this is the first study conducted in team sports with this direction, especially in the Brazilian context, thus making this unique. This prospective analysis could provide answers to certain questions in the talent identification systems and in the training process that help to mitigate the negative effects and provide more productive sport trails. Therefore, this study examined individual, task, and environmental constraints that influence the career progression of youth Brazilian elite basketball players and the probability of reaching the NBB and to determine if the association of RAE is a key factor in the career progression.

## Materials and Methods

### Design

This study presents a prospective associative strategy design (Ato et al., 2013) that analyzes, from a multifactorial perspective, the different factors that influence throughout the sport career of youth Brazilian basketball player to reach a high level (NBB).

### Sample

The sample consisted of 4,692 male players who were registered to participate in at least one U15, U17, or U22 youth Brazilian basketball championship from 2004 to 2018, all Brazilians, and were born between 1982 and 2003. The players were ranked within each category when they appeared for the first time on the database, despite having played in another category or not. Therefore, every available player registered was considered and no player was excluded; instead, all players were included. In the U15 and U17 categories, the athletes selected to represent their respective state selection teams in the Brazilian Basketball Championships organized by the CBB were included, only until 2015, and in the years 2010 and 2014, the U15 was not performed. In the U22 category, those who competed in the Basketball Development League (LDB) for their respective clubs, organized by the National Basketball League (LNB) were included, which was raised only in 2008 (the first NBB championship), and the U22 was raised for the LNB only in 2011. The CBB organized the U19 Brazilian Basketball Championships state selection teams in 2010 and 2011; these players were not included in the sample of this present study. Athletes were categorized according to their career progression in the NBB players—those who progressed in their careers from the youth categories to the high-level (NBB—Brazilian Professional Basketball League) (*n* = 452) and lower-level players—athletes who did not reach the NBB (*n* = 4,240). The lower-level category careers were defined by the progression of each player within the youth categories (U15, U17, and U22—youth state national championships) from 2004 to 2018, once, twice, or three times. The use of public data available on the Internet has been described in other studies without the need for research approval by an ethics committee (Côté et al., 2006; Werneck et al., 2016). The data were obtained according to Resolution No. 510, on April 7, 2016, from the National Council of Health, Brazil. All the research procedures were conducted in accordance with the Declaration of Helsinki.

### Variables and Procedures

Data from U15 and U17 competitions were taken from the CBB website (http://www.cbb.com.br). Data from U22 and NBB were obtained from the website of the LNB (http://www.lnb.com.br). The data set had 10,856 data points from 4,692 athletes registered in the official database of the CBB and LNB between 2004 and 2018. In order to analyze the sport career of the athlete, the category that the athlete competed (competitive level) was considered: if he played the U15, U17, or U22 or not and whether he played the NBB or not. The first and last participation of the players in a championship from 2004 until 2018 was considered a reference in order to calculate dichotomous variables from the original ones. For example, changed geographic region = 1 could be a U15 player from the south region who has changed to the southeast region at U22. In order to conduct a multifactorial analysis, a constraint-based theoretical model was utilized (Wattie et al., 2015).

### Individual Constraints

The birthdates, height, and body weight of the players were reported by teams at the moment of the competition registration. Player age was calculated by subtracting the birth year of the player from the championship year. The BioFit® software^1^ was used to assess the U15 and U17 biological maturation of the players (https://labespee.ufop.br/atletas-de-ouro). This software estimates predicted age at peak height velocity (APHV) from maturity offset—years from APHV. The following equation was used: maturity offset (years) = −7.999994 + [0.0036124 × (age × stature)] (Moore et al., 2015). The maturity status was classified as early (APHV < 13.1 years), on time (13.1 ≤ APHV ≤ 15.1 years), or late (APHV > 15.1 years) (Kozieł and Malina, 2018).

### Task Constraints

Based on the sport context, positions of the players (point guard, shooting guard, small forward, power forward, and center), competition year, and competition category (U15, U17, and U22) were obtained. Previously, the dichotomous variable was designed [played as a center (yes or no) and changed position (yes or no)] for the playing positions of the athletes. This strategy was used to identify which player position could have a significant impact in the distribution of dependent groups and latter logistic regression. The first and last competition categories of the players in a championship were recorded as their initial and final competition category from 2004 until 2018, respectively. The number of competition categories that athletes competed was created: once, twice, or three times.

### Environmental Constraints

The state and geographic regions of the teams (north, south, southeast, northeast, and midwest) were used to calculate the variables changed state (yes or no), changed region (yes or not), and played in the southeast (yes or no). In order to analyze the influence of the RAE on career progression, players born in January–March were categorized as quarter 1, April–June as quarter 2, July–September as quarter 3, and October–December as quarter 4. Semester 1 (January–June) and semester 2 (July–December) were calculated.

### Sport Performance

Regarding the sport performance, it was registered according to team performance based on the ranking of the teams in the championship (range: 1st to 24th place). The players were assigned to two categories according to the obtained results of the teams: medalists (first, second, or third places) or non-medalists (fourth place or less). They were coded as improved performance (yes or no) if the team performance in the last championship was better than in the first one.

### Statistical Analysis

Data are presented as mean ± standard deviation and percentages. First, the differences between groups were investigated with the *Student t-test*. The effect size was evaluated by *Cohen's d* (Cohen, 1992). Second, *chi-square test* (χ^2^) was used to test bivariate association between predictors and career progression. The effect size was evaluated by *phi de Cramer Coefficient* (ϕc) (Newell et al., 2014). The level of association was interpreted by the Crewson (2006). The *odds ratio* (OR) with a 95% confidence interval (95% CI) was calculated and interpreted as follows: <1.23 (very small), 1.23–1.85 (small), 1.86–2.99 (medium), and >2.99 (large) (Olivier and Bell, 2013). In the multivariate analysis, a binary logistic regression was used to examine the influence of each predictor on the probability of a youth Brazilian basketball player to reach the NBB. A backward stepwise elimination method was employed to build the model. The model fit was assessed as the model chi-square, −2 log-likelihood value, Nagelkerke's *R*^2^, and Hosmer and Lemeshow's test. The 10% cutoff limit was considered for the athlete to be classified as “NBB player.” When replacing the values of predictor variables in the formula, results >0.1 would be classified as “NBB player”; otherwise, they would be classified as “lower-level players.” The discriminant ability of the model was assessed by generating a receiver operating characteristic (ROC) curve to plot the true positive rate (sensitivity) against the false positive rate (1–specificity). An area under the curve (AUC) was calculated with an AUC of 1 (100%) representing perfect discriminant ability. Effect sizes were assessed using OR defined as the exponential of the regression coefficient *e*^*B*^. It was reported that when an OR was >1.0, it increased the odds of reaching the NBB. Conversely, when an OR was <1.0, it decreased the odds of reaching the NBB. For an OR to be significant, 95% CI would not contain the null OR of 1.0. All statistical analyses were conducted using IBM SPSS statistical software (version 24.0, IBM SPSS, Armonk, NY, USA). The statistical significance was set at *p* < 0.05.

## Results

Sample characteristics are presented in Table 1. Descriptive results obtained of level of participation of Brazilian basketball players in U15, U17, and U22 national championships are demonstrated in Table 2. Of the total sample, considering lower-level categories in the career progression, 72.2% (*n* = 3,391) played only one category, 23.3% (*n* = 1,094) played two categories, and only 4.4% (*n* = 207) played U15, U17, and U22. Considering the career of the NBB players, 9.6% of all athletes who participated in an official national championship U15, U17, or U22 from 2004 to 2018 reached NBB.

**Table 1:** Sample characteristics of youth male Brazilian basketball elite players who have participated in U15, U17, or U22 national championship between 2004 and 2018.

**Variable**	**U15** **(*n* = 2,534)**	**U17** **(*n* = 1,480)**	**U22** **(*n* = 678)**
Chronological age (years)	15.5 ± 0.7	17.3 ± 0.8	18.9 ± 1.7
APHV (years)	13.2 ± 0.5	13.5 ± 0.6	13.3 ± 0.6
Birth quartile (Q1/Q2/Q3/Q4) (%)	40.0/28.7/18.2/13.1	33.2/27.7/22.0/17.1	30.8/30.8/20.6/17.8
Height	182.7 ± 9.4	185.9 ± 10.3	192.1 ± 9.2
Weight	73.0 ± 13.0	78.6 ± 13.4	87.2 ± 13.3
Player position (PG/SG/SF/PF/C) (%)	20.6/32.6/32.9/7.6/6.3	19.5/34.2/35.3/6.9/4.1	21.5/15.1/30.9/10.9/21.6
Geographic region (N/S/SE/NE/MW) (%)	22.8/12.4/16.5/30.7/17.7	19.6/16/19.7/26.6/18.2	.0/17.6/71.1/9.0/2.4

*APHV, age at peak height velocity; Q1, 1st quartile; Q2, 2nd quartile; Q3, 3rd quartile; Q4, 4th quartile; PG, point guard; SG, shooting guard; SF, small forward; PF, power forward; C, center; N, north; S, south; SE, southeast; NE, northeast; MW, midwest*.

**Table 2:** Lower categories (U15 to U22) and NBB players' (U15–U17–U22 to NBB) career progression of youth Brazilian basketball players between 2004 to 2018 national championships.

**First national competition**	***n***	**Last national competition**			
		**U15**	**U17**	**U22**	**NBB**
U15	2,534	1,384 (54.6%)	776 (30.6%)	175 (6.9%)	168 (6.6%)
U17	1,480	–	1,229 (84.4%)	98 (6.7%)	129 (8.9%)
U22	678	–	–	523 (77.1%)	155 (22.9%)

*Thirty-one U15 athletes and 24 U17 athletes who participated in the U19 tournament were not included in the current study*.

*NBB, Novo Basquete Brasil—Brazilian professional Basketball championship*.

**Figure 1:**
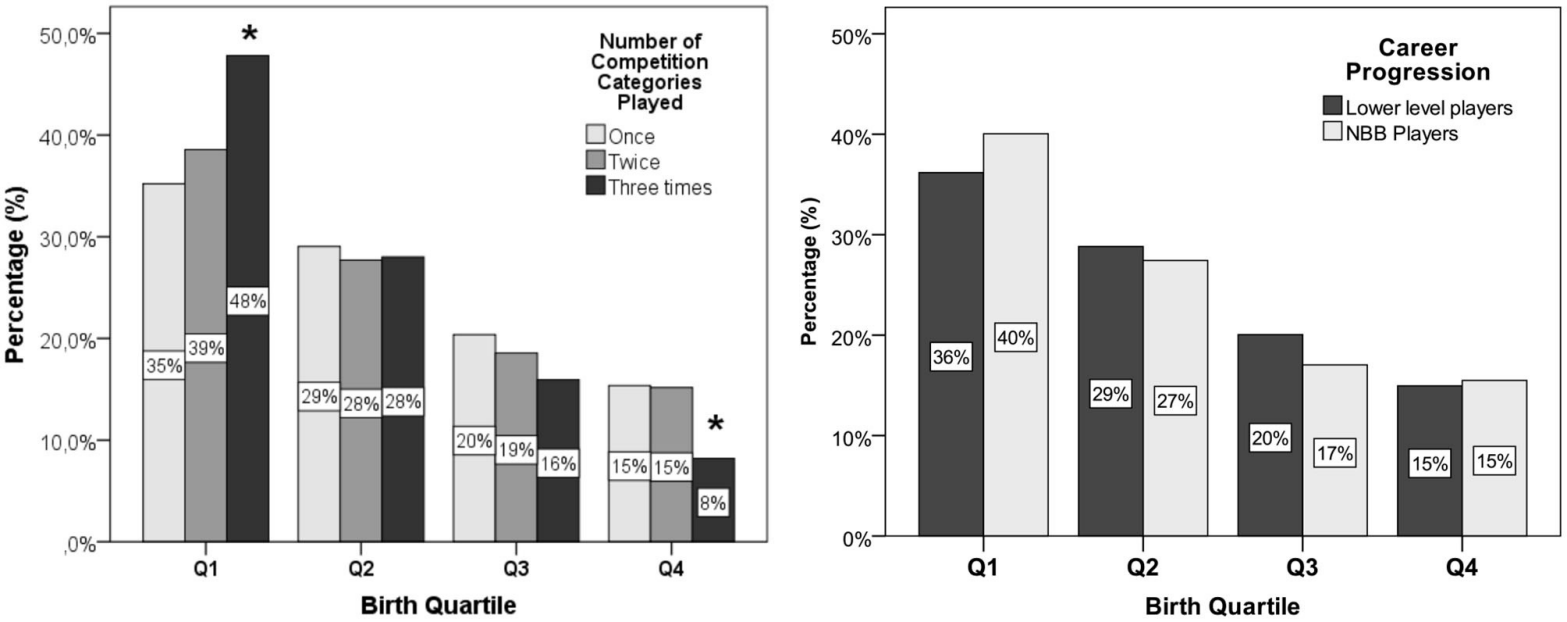
Association between birth quartile and the number of competition categories played **(left)** and career progression **(right)** of U15, U17, U22, and *Novo Basquete Brasil* (NBB) players from 2004 to 2018 championships. *p < 0.05.

As illustrated in Figure 1, there was an association between birth quartile and number of competition categories played (χ^2^ = 20.313; *p* = 0.002; ϕc = 0.05), but not with the career progression (χ^2^ = 3.896; *p* = 0.27; ϕc = 0.03). There was an overrepresentation of players born in the first quartile between athletes who were selected three times for national championship compared with the players who had played once or twice categories and lower representation of players born in the fourth quartile. However, there was no association observed in the distribution of birth quartiles between the players who reached the NBB.

**Figure 2:**
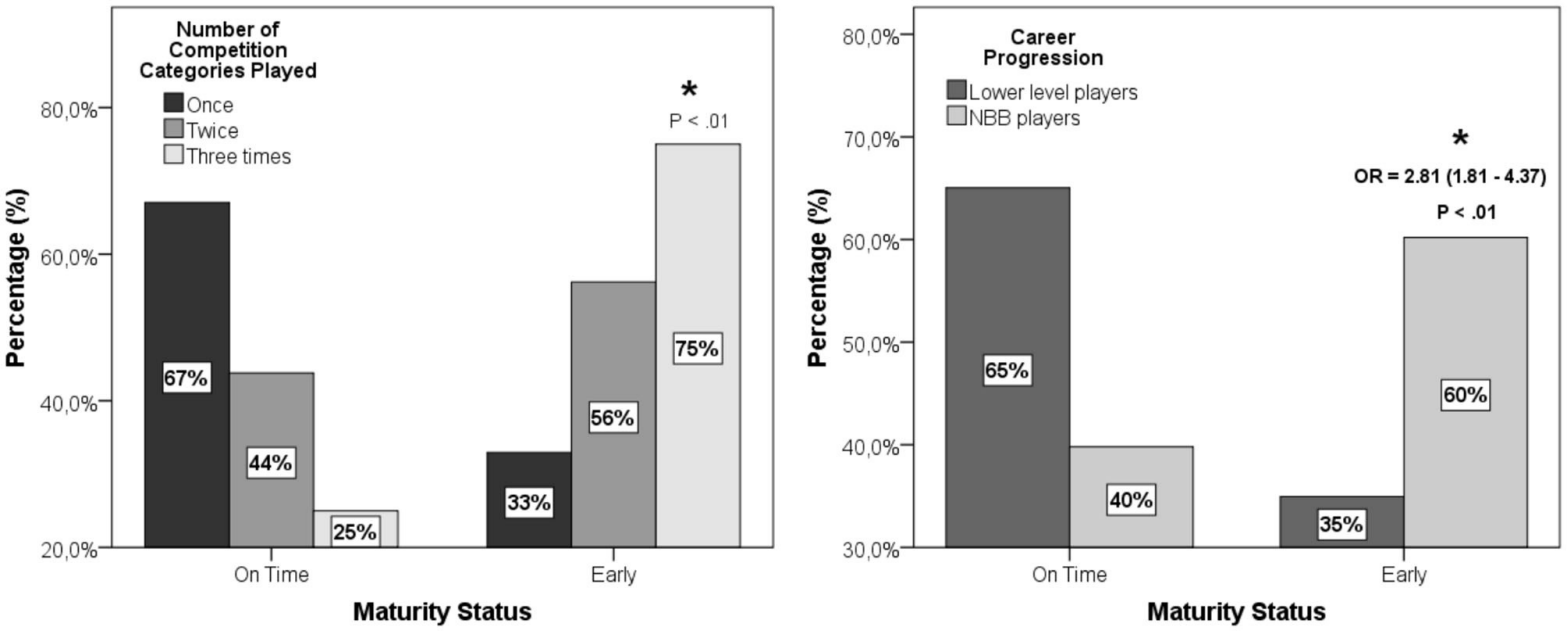
Association between maturity status and the number of competition categories played **(left)** and career progression **(right)** of U15, U17, U22, and *Novo Basquete Brasil* (NBB) players from 2004 to 2018 championships. *p < 0.05.

Regarding maturity status, early players have greater chances to progress in their careers (Figure 2). There was an overrepresentation of early players and less representation of on-time players between athletes who were selected three times for national championship (lower-level category career progression) (χ^2^ = 38.454; *p* < 0.001; ϕc = 0.24). With regard to the NBB career progression of the players, there was an overrepresentation of early players and less representation of on-time players between NBB athletes (χ^2^ = 22.335; *p* < 0.001; ϕc = 0.18). Early players were almost three times more likely to reach NBB than on-time players. It is important to show that in the sample analyzed, there were no late-maturing players.

Considering the formative category career, significant differences between NBB players and lower-level players were found (Table 3). The bivariate analysis showed that proportionally the NBB players did not compete in the U15 national championship and were selected for the first time only in the U22. Also, they were taller, were from the southeast region, were medalists, and were predominantly centers in comparison with the lower-level players. In addition, throughout their careers, NBB players were selected two to three times for national youth championships, improved their collective performance, and changed their state, region, and position on the court. There were no significant differences between groups in chronological age and birth date (quartile and semester).

**Table 3:** Mean ± standard deviation, and absolute and relative (%) frequency of variables associated with career progression of youth Brazilian elite basketball players from 2004 to 2018.

**Variable**	**NBB players**	**Lower-level players**	***P*-value**	**OR (CI 95%)**	**Effect size**
**Age at 1st championship**	16.9 ± 1.6	16.5 ± 1.6	0.26	–	0.25 (small)
**Height at 1st championship**	1.93 ± 0.09	1.84 ± 0.09	<0.001	–	1.0 (large)
**Quartile**					
Q1	181 (10.6)	1,534 (89.4)	0.10	1.18 (0.97–1.44)	Very small
Others	271 (9.1)	2,706 (90.6)			
**Semester**					
1st	305 (10.0)	2,756 (90.0)	0.293	1.12 (1.00–1.37)	Very small
2nd	147 (9.0)	1,484 (91.0)			
**Played U15**					
Yes	168 (6.6)	2,366 (93.4)	<0.001^*^	0.47 (0.40–0.57)	Medium
No	284 (13.2)	1,874 (86.8)			
**Played U17**					
Yes	143 (9.7)	2,261 (90.3)	0.860	1.02 (0.84–1.23)	Very small
No	209 (9.6)	1,979 (90.4)			
**1st category U22**					
Yes	155 (22.9%)	523 (77.1)	<0.001^*^	3.71 (2.99–4.60)	Large
No	297 (7.4%)	3,717 (92.6)			
**Number of categories competed**					
2 or 3	235 (18.1)	1,066 (81.9)	<0.001^*^	3.22 (2.65–3.93)	Large
1	217 (6.4)	3,174 (93.6)			
**Southeast region at 1st championship**					
Yes	328 (27.6)	862 (72.4)	<0.001^*^	10.36 (8.32–12.91)	Large
No	124 (3.5)	3,378 (96.5)			
**Changed state**					
Yes	203 (52.9)	181 (47.1)	<0.001^*^	18.30 (14.41–23.30)	Large
No	249 (5.8)	4,059 (94.2)			
**Changed region**					
Yes	103 (42.9)	137 (57.1)	<0.001^*^	8.83 (6.69–11.67)	Large
No	349 (7.8)	4,103 (92.2)			
**Team performance at 1st championship**					
Medalist	230 (13.7)	1,443 (86.3)	<0.001^*^	2.00 (1.65–2.41)	Medium
Not a medalist	222 (7.4)	2,797 (92.6)			
**Improved collective performance**					
Yes	125 (14.8)	719 (85.2)	<0.001^*^	1.87 (1.50–2.33)	Medium
No	327 (8.5)	3,521 (91.5)			
**Center position at 1st championship**					
Yes	61 (19.6)	250 (80.4)	<0.001^*^	2.50 (1.84–3.35)	Medium
No	391 (8.9)	3,990 (91.1)			
**Changed player position**					
Yes	265 (32.2)	558 (67.8)	<0.001^*^	9.78 (7.82–12.26)	Large
No	141 (4.6)	2,903 (95.4)			

*Line percentages are shown*;

* *statistical significant relationship, p < 0.05*.

*OR, odds ratio (95% confidence interval)*.

The individual, task, and environment constraints that influenced the probability of reaching the NBB was a combination of the following characteristics: chronologically younger and taller; played in clubs in the southeast region; selected for the first time to play U22; did not play U15; have been selected more than once to play the U15, U17, or U22 championship; and have changed playing position, club, and region over time (Table 4). About 50% of the variability in the chance to play the NBB could be explained by the model (*R*^2^ = 51.8). The model proved to be valid in the classification of the career progression of the athletes: sensitivity (87.9%) and specificity (85.5%) (Table 4).

**Table 4:** Binary logistic regression model predictive of a youth Brazilian elite basketball player to reach *Novo Basquete Brasil* (NBB).

**Predictor**	***B***	**SE**	***p***	**Exp (*B*) (95% CI)**	**Effect size**
Age at 1st championship (years)	−0.209	0.072	0.004	0.81 (0.70–0.93)	Small
Height (cm)	0.051	0.008	<0.001	1.05 (1.03–1.07)	Small
Played U15 (yes = 1)	−1.009	0.233	<0.001	0.36 (0.23–0.57)	Medium
First category U22 (yes = 1)	1.051	0.231	<0.001	2.86 (1.82–4.50)	Medium
Number of categories competed (≥2 = 1)	1.145	0.226	<0.001	3.14 (2.02–4.90)	Large
Played in the southeast (yes = 1)	1.994	0.173	<0.001	7.34 (5.23–10.31)	Large
Changed state (yes = 1)	1.588	0.216	<0.001	4.90 (3.20–7.48)	Large
Changed region (yes = 1)	0.792	0.275	0.004	2.21 (1.29–3.78)	Medium
Changed player position (yes = 1)	1.509	0.170	<0.001	4.52 (3.24–6.30)	Large
Constant	−10.423	1.871	<0.001	–	

**Figure 3:**
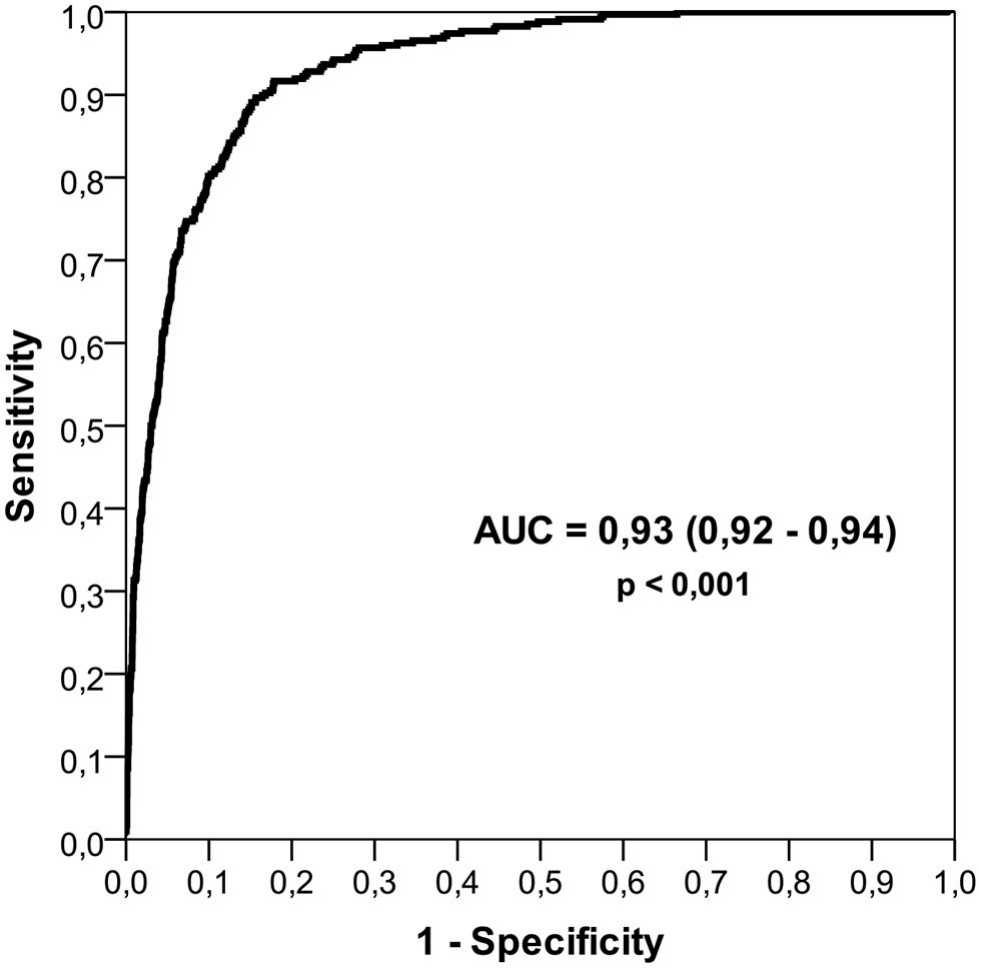
Receiver operating characteristic (ROC) curve indicating area under the curve (AUC)—ability of the model to discriminate between youth Brazilian elite player who reached *Novo Basquete Brasil* (NBB) (high performance) and those who did not.

In order to predict the probability of a youth Brazilian player in reaching the NBB, the following equation could be used: *Y* = 1/{1 + *exp* [−10.423–0.209 ^*^ age at 1st championship (years) + 0.051 ^*^ stature (cm)–1.009 ^*^ played U15 (yes = 1) + 1.051 ^*^ 1st competition category U22 (yes = 1) + 1.145 ^*^ no. categories disputed (2 or 3 = 1) + 1.994 ^*^ played in the southeast region (yes = 1) + 1.588 ^*^ changed state (yes = 1) + 0.792 ^*^ changed region (yes = 1) + 1.509 ^*^ changed playing position (yes = 1)]}. The value of Y > 0.10 is the cutoff to become an NBB player. The ROC curve demonstrated an AUC of 93%—excellent (Figure 3).

## Discussion

This study examined the association between multifactorial characteristics and career progression of youth Brazilian elite basketball players within a longitudinal and constraint-based approach. The main findings were that (1) individual characteristics—being taller and younger—increased the likelihood of reaching NBB; (2) a late selection, late specialization, and being re-selected for the youth national championship were positively associated with “NBB player's” career; (3) players were more likely to reach NBB if they were from the southeast region and who moved to other state or geographic region; (4) RAE influences lower category career, but it does not determine success; (5) early-maturing players have a greater chance to reach high performance; and (6) only 9.6% of all players from the national youth Brazilian championship U15, U17, and U22 from 2004 to 2018 reached the NBB. A logistic model displayed excellent discriminant ability between NBB players and lower-level players. These results demonstrated that a combination of individual, task, and environmental constraints influences career progression of a young Brazilian basketball player to reach the NBB.

This study is the first in modeling the career progression of youth Brazilian basketball players. The model observed a higher probability of reaching the NBB in athletes with the following characteristic combination: chronologically younger and taller, who play for clubs in the southeast region, are selected for the first time to play U22, not playing U15 national championship, have been re-selected to play the youth national basketball championships, and over time have changed playing position, club, and region. The current understanding is that multidisciplinary and dynamic approach must be used to assess sport potential (Ribeiro Júnior et al., 2019) and development (Sáenz-López et al., 2005) of young basketball players. Talent development and expertise must be taken into account with a wide range of factors (Gulbin et al., 2013; Hambrick et al., 2016).

Regarding individual constraints, athletes who reached the NBB are taller and younger than lower-level players in the first competition category, after controlling for possible confounders. The odds of reaching NBB increase 5% for each centimeter in height and lower 23% for each chronological year. Previous studies demonstrated that stature is a key factor for high performance (Ribeiro Júnior et al., 2020; Zarić et al., 2020), as well as the selection (Torres-Unda et al., 2013; Baxter-Jones et al., 2020; Ribeiro Júnior et al., 2020), in order to continue participating in basketball (Baxter-Jones et al., 2020). Additionally, young basketball players from 10.5 to 15.5 years of age over the course of 2 years found that the tallest players are more likely to be selected and/or promoted, regardless of their low functional skills (Soares et al., 2020). Body height have had the highest priority during the selection process and when establishing an in-court position (Zarić et al., 2020) In this study, NBB players were 1 year younger but were selected to compete at the national level 1 year after the lower-level players. These results confirm the findings of Leite and Sampaio (2012), where it was demonstrated that when starting to compete over the U14, the athletes are less likely to reach the highest levels. In general, younger athletes have greater development potential, and when this potential is associated with the process of full- and long-term training, it favors career progression up to high-performance levels (Till et al., 2016).

Regarding task constraints, late selection, being re-selected to youth national championship, and late specialization were positively associated with the career of NBB players. Only 9.6% of all players from the national youth Brazilian championship U15, U17, and U22 from 2004 to 2018 reached NBB. Logistic model displayed excellent discriminant ability between NBB players and lower-level players. Our results are similar to the Spanish basketball (Sáenz-López et al., 2006; Ibáñez et al., 2010), question pyramid-based model of talent development (Bailey and Collins, 2013) and suggest that professional players emerged from repeated procedures from selection and disqualification procedures from talent identification process throughout the specialization as an expert (Güllich, 2014). Regarding the first competition category, the chances of playing NBB increase from the U15 (6.6%) and U17 (8.9%) until U22 (22.9%). Being selected for U15 championship diminishes the probability of playing the NBB. According to Güllich and Emrich (2014) and Cunha et al. (2017), a good performance in the youth categories is not a guarantee of career progression. An early start-age for training and competition favored early adolescent success but did not contribute to individual differences in the success achieved at a senior age (Güllich and Emrich, 2014).

In the present study, the highest percentage of selected athletes for NBB was 22.9% of the athletes who played U22 championship. Hence, the athletes who played the U22 championship increased the chances of playing NBB by 18 times than did the athletes who did not play the U22. The closer the category is to the adult high-performance stage, the greater the chances are of reaching a higher level (Sáenz-López et al., 2006; Feu et al., 2008; Ibáñez et al., 2010). A study conducted with the Spanish basketball players showed that 39.6% of the athletes selected in the junior teams reached the professional league (Subijana and Lorenzo-Calvo, 2018). Findings from Sáenz-López et al. (2006) study demonstrated that in the U22 category, 24% reached the adult category and 94% were established in the professional league, therefore confirming that athletes who have a more active participation in the U22 tend to participate also in the adult category. The U22 category is a way to develop their sporting potential. Even though this athlete was not selected in the previous stages, he remained in the development process, and he became more mature and more experienced, with better technical and tactical skills.

Re-selection seems to be an important factor for a career progression in the youth Brazilian basketball. In the present study, the odds of reaching the NBB are three times greater for an athlete who has played two or more competition categories. These results suggest that the athletes who really combine long-term sport potential and current performance make experience a key factor to keep them in high-performance levels. In soccer, athletes who progressed to professional status at 16 years of age accumulated more hours per year in soccer playing activities between 6 and 12 years of age than did those who did not progress (Ford et al., 2009). These findings reinforce the importance of a deliberate play and appropriate practice according to the reality of the individual, which provides stability between the process of developing sports potential and competitive performance (Moxley and Towne, 2015). In European basketball youth national teams, 75% of male players were re-selected the following year, but the chance of re-selection until age 20 is lower for players initially selected at age 16 compared with all other ages (Kalén et al., 2020). Consequently, the re-selection is not a prerequisite to reach high performance. The study performed by Gulbin et al. (2013) investigating 256 elite athletes across 27 different sports found that non-linear careers were experienced by the majority of the athletes and linearity of junior to senior competition transition was observed in <7% of the cases.

Considering the playing position at the first competition category, centers (usually with greater stature) have seven times more chances to play NBB than point guards, small forwards, forwards, and power forwards. At the same time, it cannot be said that when they have reached the NBB, these athletes who were considered “centers” in the youth categories remain centers in the adult level. In general, the high stature becomes a prerequisite to play the game, although it must be associated with specific skills to perform various functions in the game (Zarić et al., 2020). The findings of the present study pointed out that athletes who changed their playing position throughout their career present up to 4.5 more chances of reaching the NBB. Late specialization seems to be a key to success. Elite athletes were shown to intensify their training regime during late adolescence (Moesch et al., 2011). The multilateral stimulus during early ages is very important to the acquisition and development of fundamental movement skills in basketball (Arede et al., 2019). Thus, universal talent development (without specializing by position) should be a rule and not an option. The optimal career path is a combination between amount, quality, and when training regimes occur (Moesch et al., 2011).

Regarding environmental constraints, some studies have assessed whether “where” an athlete is born influences their likelihood of playing a professional team sport—this is called *birthplace effect*. A comparative analysis suggested that sociocultural factors, like place of birth, contribute more to the achievement of an elite level of sport performance than does relative age, for example (Côté et al., 2006; Baker et al., 2009). The quality of evidence that birthplace offers an advantage in regard to the development of a super-elite performance in sport is high to moderate, in spite of the fact that birthplace itself may not be as critical as the early development place. It is important to study and understand the effects of how the environment and neighborhoods in which the prospect athlete was raised can affect their future performance (Rees et al., 2016).

Our results showed that athletes from the southeast region and who moved to more developed Brazilian basketball centers all enhanced their chances of reaching the NBB. Athletes who had played in the southeast during the first competition category were the most important predictor for the “NBB players” career (large effect size). Besides, athletes who changed from one state to another and those that changed to other geographic region had better chances of reaching the NBB in relation to their peers. In Brazil, high-performance sport is centered in the southeast—a region of greater economic power in the country. The results of NBB 2014/2015 season indicated that there is a predominance of clubs from São Paulo state in the training of athletes, especially the city of Franca (Cunha et al., 2017). However, this centralized sport scenario did not favor the development of the Brazilian basketball (Beneli, 2018). A talented player needs to have an early exposure in an environment with more opportunities to develop to their full potential in basketball. Policy makers and practitioners must take into consideration the contextual factors when designing talent selection and development process.

In the present study, RAE influences the lower-level category career, but it does not determine success. The re-selected players who participated in the U15, U17, and U22 championship were 48% from Q1 and only 8% from Q4. The RAE relates to selection bias toward individual athletes born earlier in the year—chronologically older in the same competition category. This is a prevalent phenomenon in the Brazilian basketball (Oliveira et al., 2017, 2019; Ribeiro Júnior, 2020; Ribeiro Júnior et al., 2020). The coaches tend to select athletes born in the first quartile/semester in the youth categories, and this study confirms their influence on immediate success. However, RAE is not decisive for reaching the highest level. The de la Rubia et al. (2020) systematic review observed that the presence of the RAE is related to the competitive performance in the short term; in the contrary, in the long term, this effect is reverse in team sports. In the European youth national teams, the re-selection process is 20–25% greater for players born in the fourth quarter up until age 20 than for the players born in the first quarter (Kalén et al., 2020).

When observing the birth date in the Brazilian basketball, those born in the first months of the year are not necessarily those who played the NBB. For example, two younger athletes, even born in the same day, may have markedly different developmental experiences (Wattie et al., 2015). The direct and indirect RAE is on performance diagnostics during the talent identification process, especially body size and motor performance; and it is less evident in technical skills (Votteler and Höner, 2014). Athletes selected and competing in the youth categories, especially U15 and U17, are in general selected focusing on immediate results from temporary advantages provided by the RAE, while not favoring the development of the sport potential of Brazilian basketball. Young athletes may depart the sport prior to full maturity without having the opportunity to nurture their skills and inherent interest (Cobley et al., 2009). It is important to identify and develop talents providing a greater equality of opportunities to all athletes regardless of the birth month (de la Rubia et al., 2020). The search for immediate results by the scouters/coaches (Subijana and Lorenzo-Calvo, 2018), competitive environment of natural selection (Cobley et al., 2009; de la Rubia et al., 2020), lack of knowledge about the consequences of RAE (Hancock et al., 2013), and how much the lack of this knowledge can hinder the long-term training process are important aspects when overestimated or ignored. Coaches, scouters, and managers have to pay special attention to the RAE phenomenon in the selection processes.

There is a constraint-based developmental system model for RAE in sport with hypothetical causal components and interactive contribution to each individual, task, and environment constraint (Wattie et al., 2015). Another theoretical model proposes that social agents (parents, coaches, and athletes) have the largest influence on RAE (Hancock et al., 2013). One of the possible explanations of RAE, especially in team sports, is the hypothesis of biological maturation (Baxter-Jones et al., 2020). Our results showed that early-maturing players have three times more chance to reach high performance than on-time players. Maturational status seems to be a key aspect in physiological performance and selection in elite male basketball players (Arede et al., 2019). Coaches rating long-term potential of early maturing players as greater cause late-maturing athletes to have an increased risk of de-selection (Cripps et al., 2016). Early maturing players are frequently considered the “best” due to the advantages of strength, coordination, speed, and power, which favor being chosen to represent teams in their respective categories.

According to Subijana and Lorenzo-Calvo (2018), the athletes who participate in regional, state, and national teams have more time for deliberate practice and gain more learning experience. This fact may keep them during the career progression process, increasing the chances of these athletes to reach the high level contrary to the late-maturing ones. Nevertheless, future researches should observe the profile of these advanced athletes who reached the NBB in terms of their high-performance career, their continuity, and their competitive importance at this level, in order to solve doubts, if these advanced athletes have a representative career in the NBB, or just reached the high adult level of the Brazilian basketball, but do not remain competing. In rugby, late-maturing athletes appear to show more progression and “catch up” to the early-maturing player during adolescence over a 2-year period (Till et al., 2014). Coaches should be educated that slower responders may possess as much or more ability as fast responders to training programs. Both late developers and slower responders should be maintained in the process (Pearson et al., 2006).

The analysis presented in this study used a longitudinal and constraint-based approach to examine a number of available parameters. However, it is important to acknowledge that career progression of youth Brazilian basketball may be influenced by many others factors that are beyond the scope of this study. For example, motor performance, technical skills, tactical skills, psychological and sociological indicators, and individual performance are likely to influence the selection process and career progression of young basketball players.

Furthermore, individual, task, and environmental constraints should be used in a combined and longitudinal way for a better understanding of sport potential and career progression of young Brazilian basketball players. It is important to highlight not only the athletes who present immediate results in the initial stages of training process. The selection of athletes based on criteria, such as RAE, biological maturation, and high stature at the youth categories may diminish the opportunities of athletes with high sport potential in not participating in the national youth championships. Coaches need to assess the biological maturation of each player in order to minimize the risk of mistaken judgments and errors in the selection process, as well as the early exclusion of high potential young athletes. Athletes who show a late growth may not have the opportunity to develop their skills due to less playing time and participation in national-level championships. Thus, new opportunities for selection and re-selection of younger and late-maturing athletes should be given because they have a doubly temporary disadvantaged (Rubajczyk et al., 2017).

## Limitations

First, the lack of sequences in the national competitions could provide some missing data that would provide more relevant information in the all level/category evaluated. Second, we did not consider a group of minors (players who played in above category) that could be addressed to control the chronological age and competitive level. This information is really important, although it was not the focus in the main objective of this present study; and it needs further investigation. Third, the database did not contain the individual performance in competition, and it was not provided from the organizers of the tournaments. This kind of data would be very important to present not only the presence of player in career progression but also the real impact of this participation on court.

## Conclusion

The main study findings were a combination of individual, task, and environmental characteristics that influence the sport career of a young Brazilian basketball player in reaching excellence. The taller younger players, not selected for the U15 championship, who played U22 championship as the first competition category and are from the southeast region, while having played two or three youth national championships, not specializing too early in the playing position, and have moved to more developed Brazilian basketball centers all enhanced their chance of reaching the NBB. The birth dates of the athletes influence selection and re-selection to youth national championship, but they do not determine the “NBB players” career progression. In contrast, biological maturation is a key factor to reaching a higher performance level. Only 10% of the players in the youth Brazilian basketball championships U15, U17, and U22 reached the NBB from 2004 to 2018. These results present a holistic viewpoint from talent identification through the expert performance career and have the ability to assist in decisions for optimizing the career progression of youth Brazilian basketball players.

## Data Availability Statement

The original contributions presented in the study are included in the article/supplementary material, further inquiries can be directed to the corresponding author/s.

## Ethics Statement

Ethical review and approval was not required for the study on human participants in accordance with the local legislation and institutional requirements. Written informed consent from the participants' legal guardian/next of kin was not required to participate in this study in accordance with the national legislation and the institutional requirements.

## Author Contributions

DR, FW, HO, SI, and JV: conceptualization, formal analysis, methodology, project administration, supervision, validation, visualization, and writing—review and editing. DR, HO, and FW: investigation, resources, and writing—original draft preparation. All authors contributed to the article and approved the submitted version.

## Conflict of Interest

The authors declare that the research was conducted in the absence of any commercial or financial relationships that could be construed as a potential conflict of interest.

## Footnotes

^1^Projeto Atletas de Ouro®. *BioFit*®–*Avaliação da Maturação Biológica*. Available online at: https://labespee.ufop.br/atletas-de-ouro (accessed September 1, 2020).
